# Supplementary material for: The impact of policy restrictions and mobility changes on excess mortality during the COVID-19 pandemic in The Netherlands, 2020–2022
Source: PLoS One. 2026 Feb 9;21(2):e0322350. doi: 10.1371/journal.pone.0322350 (PMC12885301; doi:10.1371/journal.pone.0322350)
Supplement: S1 File — Additional analyses and robustness checks. (PDF) [file pone.0322350.s001.pdf]

Supporting Information

Policy restrictions as a function of COVID-19 cases and deaths

To confirm that information about the pandemic influenced the likelihood that policy measures will be imposed or lifted, we look at the stringency of the policy restrictions in force as a function of the pandemic-related information. Models S1a and S1b in Table S1 analyse *the level* of policy restrictions in place as a function of the (logged) number of the newly-registered COVID-19 cases and the number of deaths (in the preceding week). Models S1c and S1d analyse *the change* in the policy stringency index as a function of the growth rate in COVID-19 cases[1] and deaths. As the data are aggregated at the weekly level, we expect that the number of cases in the same week and the number of deaths in the preceding week are most relevant for the level of policy restrictions in force.

Table S1 shows the results of two linear regression models for each of the two outcomes of interest (levels and changes in the policy): one *with* and one *without* interactions between the predictors and the three waves of the pandemic in the period 2020-2022. In all four models the standard errors are clustered at the level of the province, because there is no variation in the policy data at that level. We also estimate the models only at the country level, reaching very similar results.

As expected, both the number of cases and the number of deaths are significant predictors of the stringency of the public policy in place in the subsequent week (Models S1a and S1b). The effect of the number of cases was strongest (largest coefficient) during the first wave, but declined significantly in the subsequent two waves. At the same time, the effect of the number of deaths remained constant during the first two waves and even increased during the third wave. Fig S1 provides an illustration.

---

[1] The growth rate is calculated as the log in the number of new COVID-19 cases registered in week *x* minus the log of the number of new COVID-19 cases registered in week *x-1*. We add one case to all weeks to avoid the log function returning -Infinity in the very few weeks with zero cases.

Table S1. **Policy stringency as a function of COVID-19 cases and deaths, per COVID-19 wave.**

	**Level of Policy stringency index**		**Change in Policy stringency index**	
	**Model S1a**	**Model S1b**	**Model S1c**	**Model S1d**
(Intercept)	3.43 [2.99, 3.87] p<0.01 ***	3.16 [2.34, 3.98] p<0.01 ***	0.13 [0.11, 0.15] p<0.01 ***	0.09 [0.05, 0.12] p<0.01 ***
COVID-19 cases<br>- log (number)<br>- growth rate	0.35 [0.26, 0.43] p<0.01 ***	0.45 [0.25, 0.64] p<0.01 ***	0.31 [0.25, 0.36] p<0.01 ***	0.57 [0.43, 0.70] p<0.01 ***
COVID-19 deaths<br>- number (lag 1)<br>- growth rate	0.01 [0.00, 0.02] p<0.01 **	0.01 [0.00, 0.01] p=0.02 *	0.01 [0.00, 0.01] p<0.01 ***	0.01 [0.01, 0.01] p<0.01 ***
Second wave (indicator)	−0.54 [−0.88, −0.21] p<0.01 **	−0.03 [−0.96, 0.90] p=0.95	−0.18 [−0.20, −0.17] p<0.01 ***	−0.13 [−0.16, −0.09] p<0.01 ***
Third wave (indicator)	−4.32 [−4.84, −3.80] p<0.01 ***	−3.42 [−4.19, −2.65] p<0.01 ***	−0.16 [−0.18, −0.15] p<0.01 ***	−0.12 [−0.16, −0.09] p<0.01 ***
COVID-19 cases × Second wave		−0.13 [−0.30, 0.04] p=0.12		−0.43 [−0.58, −0.27] p<0.01 ***
COVID-19 cases × Third wave		−0.21 [−0.37, −0.05] p=0.01 *		−0.52 [−0.66, −0.37] p<0.01 ***
COVID-19 deaths × Second wave		0.00 [0.00, 0.01] p=0.33		0.00 [−0.01, 0.00] p<0.01 ***
COVID-19 deaths × Third wave		0.05 [0.03, 0.06] p<0.01 ***		0.00 [−0.01, 0.00] p=0.05 +
Num.Obs.	*1644*	*1644*	1644	*1644*
R2 Adj.	*0.624*	*0.639*	0.121	*0.172*

*The numbers show the unstandardized coefficients from linear regression models, which indicate the implied change in the Policy stringency index for a one-unit change in the covariate. Standard errors are clustered at the level of provinces (N=12). 95% Confidence intervals are reported in the square brackets. Significance levels of p values: *** < 0.001; ** < 0.01; *<0.05; +<0.10. The precise p values are printed when >0.01.*

The growth rate of cases and deaths also predict changes in the policy stringency index. The same pattern of declining effect for cases but increasing effect of deaths during the third wave can be observed in Models S1c and S1d.

The results are very similar when we estimate these models on national-level data only. However, due to the high correlation between the log-transformed COVID-19 cases and deaths, when both are entered in the model, the coefficient for cases is not significant, while the one for deaths is. The results are also robust to using to applying the inverse hyperbolic sin transformation instead of the logarithmic one.

***Fig S1.* Marginal effects (point estimates and 95% confidence intervals) of the number of COVID-19 cases and deaths on the level of Policy stringency in The Netherlands, across the first three waves of the pandemic (2020-2022)**

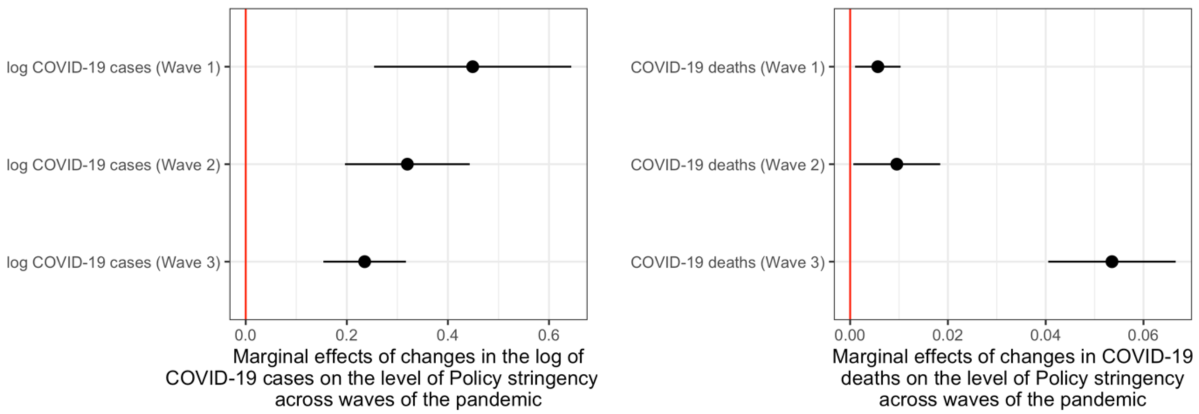

The figure shows the predicted effects of one-point increases in the log of COVID-19 cases (left panel) and COVID-19 deaths (right panel) on the level of Policy stringency during each of the three stages of the pandemic in the period 2020-2022 in The Netherlands. One-point increases in the log of the number of cases and deaths correspond approximately to doubling the number of cases and deaths. Results based on Model S1b in Table S1.

Table S2. **Changes in mobility as a function of policy stringency, COVID-19 cases and deaths, and additional covariates, including interaction effects with pandemic waves.**

	**Model S2a Places of work**	**Models S2b Public transport hubs**	**Model S2c Grocery shops and markets**	**Model S2d Retail and recreation**	**Model S2e Residential places**
Policy stringency	−6.20 [−6.90, −5.50] p<0.01 ***	−7.36 [−8.25, −6.47] p<0.01 ***	−3.19 [−3.92, −2.47] p<0.01 ***	−7.86 [−8.98, −6.74] p<0.01 ***	2.04 [1.88, 2.19] p<0.01 ***
COVID-19 cases (log, lag 1)	−0.13 [−1.02, 0.77] p=0.78	−2.25 [−3.38, −1.12] p<0.01 ***	0.75 [−0.18, 1.67] p=0.11	0.26 [−1.17, 1.68] p=0.72	0.42 [0.23, 0.61] p<0.01 ***
COVID-19 deaths (log, lag 1)	0.44 [−0.47, 1.34] p=0.35	1.14 [−0.01, 2.29] p=0.05 +	−0.99 [−1.93, −0.05] p=0.04 *	−2.00 [−3.45, −0.55] p<0.01 **	0.07 [−0.13, 0.26] p=0.51
Second wave	−25.27 [−30.07, −20.48] p<0.01 ***	−24.52 [−30.61, −18.43] p<0.01 ***	−15.58 [−20.51, −10.65] p<0.01 ***	−3.92 [−11.53, 3.70] p=0.31	4.87 [3.85, 5.90] p<0.01 ***
Third wave	−24.89 [−29.24, −20.55] p<0.01 ***	−7.14 [−12.63, −1.65] p=0.01 *	5.00 [0.51, 9.50] p=0.03 *	−8.99 [−15.92, −2.07] p=0.01 *	3.74 [2.80, 4.67] p<0.01 ***
Public holidays	−11.38 [−12.84, −9.91] p<0.01 ***	−6.06 [−7.91, −4.21] p<0.01 ***	−6.65 [−8.17, −5.13] p<0.01 ***	−9.76 [−12.10, −7.42] p<0.01 ***	2.02 [1.70, 2.34] p<0.01 ***
Max. temperature	−0.18 [−0.25, −0.10] p<0.01 ***	0.58 [0.49, 0.68] p<0.01 ***	0.53 [0.45, 0.61] p<0.01 ***	1.49 [1.37, 1.61] p<0.01 ***	−0.16 [−0.18, −0.14] p<0.01 ***
Policy stringency × Second wave	3.80 [2.95, 4.65] p<0.01 ***	5.34 [4.27, 6.42] p<0.01 ***	1.91 [1.02, 2.79] p<0.01 ***	1.93 [0.57, 3.30] p<0.01 **	−1.29 [−1.48, −1.11] p<0.01 ***
Policy stringency × Third wave	4.30 [3.53, 5.07] p<0.01 ***	4.86 [3.88, 5.84] p<0.01 ***	3.58 [2.78, 4.39] p<0.01 ***	5.59 [4.36, 6.83] p<0.01 ***	−1.29 [−1.46, −1.12] p<0.01 ***
COVID-19 cases × Second wave	2.40 [1.38, 3.43] p<0.01 ***	2.55 [1.25, 3.85] p<0.01 ***	1.15 [0.09, 2.21] p=0.03 *	−0.15 [−1.78, 1.48] p=0.86	−0.46 [−0.68, −0.24] p<0.01 ***
COVID-19 cases × Third wave	0.44 [−0.52, 1.40] p=0.37	0.18 [−1.03, 1.39] p=0.78	−2.72 [−3.71, −1.72] p<0.01 ***	−0.97 [−2.50, 0.56] p=0.21	−0.20 [−0.41, 0.00] p=0.05 +
COVID-19 deaths × Second wave	−2.82 [−3.99, −1.66] p<0.01 ***	−4.69 [−6.16, −3.21] p<0.01 ***	−0.91 [−2.12, 0.30] p=0.14	−0.16 [−2.02, 1.70] p=0.86	0.74 [0.49, 0.99] p<0.01 ***
COVID-19 deaths × Third wave	−0.19 [−1.31, 0.93] p=0.74	−0.58 [−2.00, 0.83] p=0.42	2.52 [1.36, 3.68] p<0.01 ***	0.97 [−0.82, 2.76] p=0.29	0.08 [−0.17, 0.32] p=0.54
Num.Obs.	1626	1622	1630	1628	1632
R2 Adj.	0.584	0.820	0.700	0.820	0.892

The numbers show the unstandardized coefficients from linear regression models, which indicate the implied change on Mobility (defined as the percentage change in the presence of people compared with a baseline period in the pre-pandemic period in early 2020 in particular types of places) for a one-unit change in the covariate. The models include indicators at the province level (N=12), as well as controls for the demographic structure of the provinces (share of 65+, share of women, share of low-income households, share of 1st generation immigrants). 95% Confidence intervals are reported in

*the square brackets. Significance levels of p values: *** < 0.001; ** < 0.01; *<0.05; +<0.10. The precise p values are printed when >0.01.*

***Fig S2* Marginal effects (point estimates and 95% confidence intervals) of Policy stringency on Mobility changes in The Netherlands, across the first three waves of the pandemic (2020-2022)**

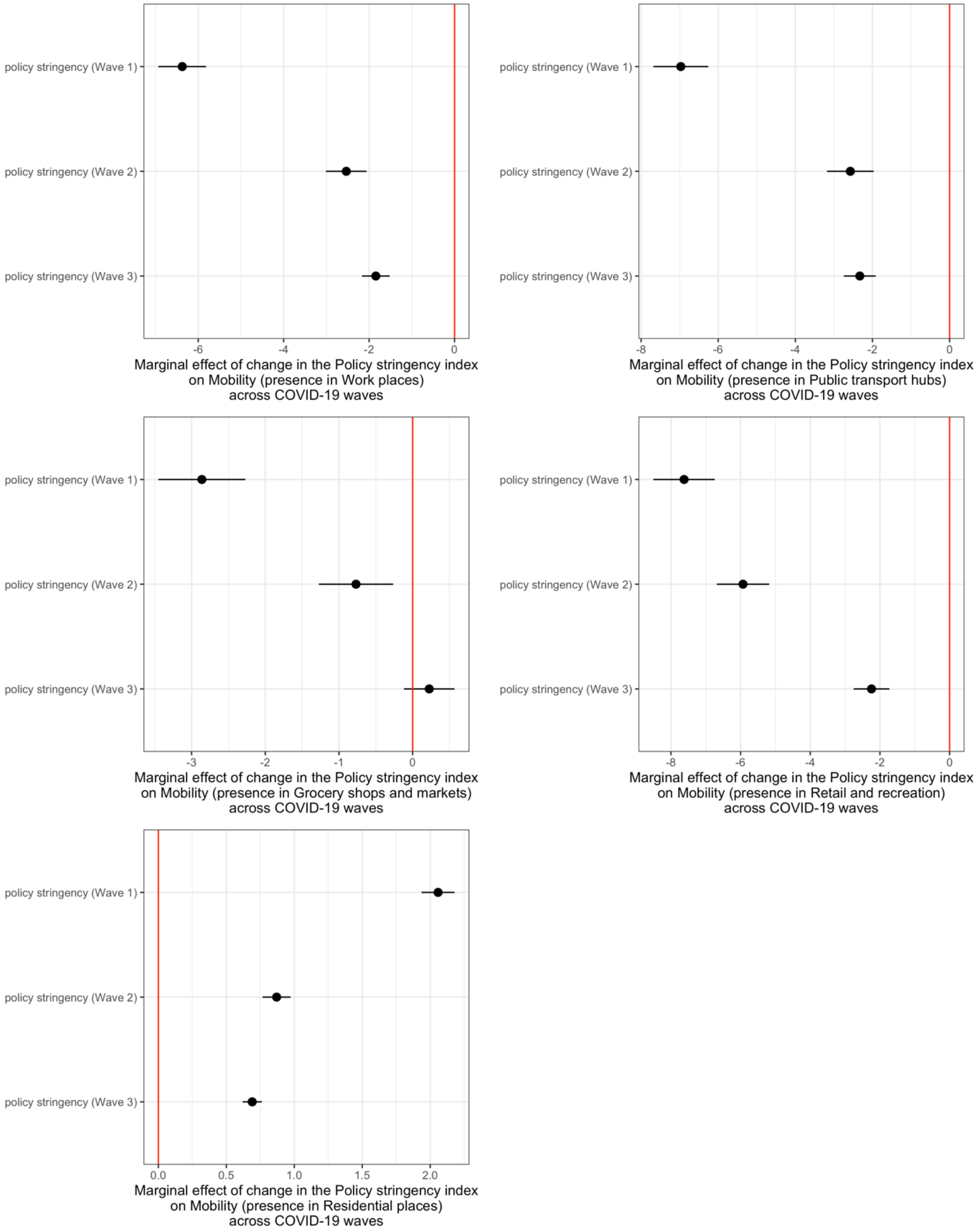

The figure shows the predicted marginal effects ((point estimates and 95% confidence intervals) of one-point increases in the Policy stringency index on Mobility (defined as the percentage change in the presence of people compared with a baseline period in the pre-pandemic period in early 2020) in five types of places (Work places, Public transport hubs, Grocery shops and markets, Retail and recreation, and Residential places) depicted in the five panels of the figure, during each of the three waves of the pandemic in The Netherlands. Results based on variations of the models reported in Table S2.

Table S3. **Number of registered COVID-19 cases (logged) as a function of policy stringency, changes in mobility and additional covariates, including interaction effects with pandemic waves.**

	**Model S3a COVID-19 cases (log)**	**Model S3b COVID-19 cases (log)**	**Model S3c COVID-19 cases (log)**	**Model S3d COVID-19 cases (log)**	**Model S3e COVID-19 cases (log)**	**Model S3f COVID-19 cases (log)**
COVID-19 cases (log, lag 1)	0.86 [0.84, 0.88] p<0.01 ***	0.85 [0.83, 0.88] p<0.01 ***	0.86 [0.84, 0.89] p<0.01 ***	0.86 [0.84, 0.88] p<0.01 ***	0.85 [0.83, 0.87] p<0.01 ***	0.86 [0.84, 0.88] p<0.01 ***
Public holidays	−0.01 [−0.10, 0.09] p=0.91	0.01 [−0.09, 0.10] p=0.91	0.01 [−0.09, 0.10] p=0.91	−0.00 [−0.10, 0.09] p=0.93	−0.01 [−0.10, 0.08] p=0.81	−0.00 [−0.10, 0.09] p=0.92
Max. temperature	−0.03 [−0.04, −0.03] p<0.01 ***	−0.03 [−0.04, −0.03] p<0.01 ***	−0.03 [−0.04, −0.03] p<0.01 ***	−0.03 [−0.04, −0.03] p<0.01 ***	−0.03 [−0.03, −0.02] p<0.01 ***	−0.03 [−0.04, −0.03] p<0.01 ***
Second wave	0.57 [0.31, 0.82] p<0.01 ***	0.66 [0.39, 0.93] p<0.01 ***	0.58 [0.32, 0.84] p<0.01 ***	0.69 [0.42, 0.96] p<0.01 ***	0.76 [0.45, 1.06] p<0.01 ***	0.57 [0.30, 0.83] p<0.01 ***
Third wave	−0.46 [−0.69, −0.24] p<0.01 ***	−0.39 [−0.63, −0.16] p<0.01 **	−0.44 [−0.67, −0.21] p<0.01 ***	−0.61 [−0.85, −0.36] p<0.01 ***	−0.54 [−0.79, −0.29] p<0.01 ***	−0.46 [−0.69, −0.23] p<0.01 ***
Policy stringency	−0.16 [−0.19, −0.13] p<0.01 ***	−0.12 [−0.16, −0.07] p<0.01 ***	−0.10 [−0.14, −0.05] p<0.01 ***	−0.16 [−0.19, −0.12] p<0.01 ***	−0.20 [−0.24, −0.15] p<0.01 ***	−0.15 [−0.21, −0.10] p<0.01 ***
Policy stringency × Second wave	−0.04 [−0.08, 0.01] p=0.09 +	−0.07 [−0.12, −0.01] p=0.02 *	−0.10 [−0.16, −0.04] p<0.01 ***	−0.07 [−0.12, −0.02] p<0.01 **	−0.07 [−0.13, −0.00] p=0.04 *	−0.04 [−0.11, 0.03] p=0.27
Policy stringency × Third wave	0.16 [0.13, 0.20] p<0.01 ***	0.12 [0.07, 0.17] p<0.01 ***	0.11 [0.06, 0.16] p<0.01 ***	0.17 [0.13, 0.21] p<0.01 ***	0.21 [0.16, 0.26] p<0.01 ***	0.16 [0.10, 0.22] p<0.01 ***
Work (lag2)		0.01 [0.00, 0.01] p=0.03 *				
Second wave × work.lag2		−0.00 [−0.01, 0.01] p=0.75				
Third wave × work.lag2		−0.01 [−0.01, 0.00] p=0.06 +				
Transport (lag 2)			0.01 [0.00, 0.01] p<0.01 ***			
Second wave × transport.lag2			−0.01 [−0.01, −0.00] p<0.01 **			
Third wave × transport.lag2			−0.01 [−0.01, −0.00] p=0.02 *			
Grocery (lag 2)				0.00 [−0.00, 0.01] p=0.52		

	**Model S3a COVID-19 cases (log)**	**Model S3b COVID-19 cases (log)**	**Model S3c COVID-19 cases (log)**	**Model S3d COVID-19 cases (log)**	**Model S3e COVID-19 cases (log)**	**Model S3f COVID-19 cases (log)**
Second wave x grocery.lag2				−0.01 [−0.02, −0.00] p=0.02 *		
Third wave x grocery.lag2				0.00 [−0.01, 0.01] p=0.73		
Retail (lag 2)					−0.01 [−0.01, −0.00] p<0.01 **	
Second wave × retail.lag2					−0.00 [−0.01, 0.00] p=0.62	
Third wave × retail.lag2					0.00 [−0.00, 0.01] p=0.14	
Residence (lag 2)						−0.00 [−0.02, 0.02] p=0.90
Second wave × residence.lag2						0.00 [−0.03, 0.03] p=0.98
Third wave × residence.lag2						0.00 [−0.03, 0.03] p=0.93
Num.Obs.	1632	1626	1622	1630	1628	1632
R2 Adj.	0.961	0.961	0.961	0.961	0.962	0.961

The numbers show the unstandardized coefficients from linear regression models, which indicate the implied change on the log of the number of COVID-19 cases for a one-unit change in the covariate. The models include indicators at the province level (N=12), as well as controls for the demographic structure of the provinces (share of 65+, share of women, share of low-income households, share of 1st *generation immigrants). 95% Confidence intervals are reported in the square brackets. Significance levels of p values: *** < 0.001; ** < 0.01; *<0.05; +<0.10. The precise p values are printed when >0.01.*

Table S4. **Linear regression models of excess mortality (the log of IRR, or the Incidence Rate Ratio), including interaction effects with pandemic waves and province**

	**Model S4a**<br>**log(IRR)**	**Model S4b**<br>**log(IRR)**
Public holidays	0.204 [0.170, 0.237]<br>p<0.001 ***	0.215 [0.181, 0.248]<br>p<0.001 ***
Minimum temperature	0.013 [0.005, 0.022]<br>p=0.003 **	0.017 [0.008, 0.025]<br>p<0.001 ***
Maximum temperature	0.000 [−0.010, 0.010]<br>p=0.997	−0.003 [−0.013, 0.007]<br>p=0.535
Policy stringency (lag 5)	−0.047 [−0.056, −0.039]<br>p<0.001 ***	−0.012 [−0.023, −0.001]<br>p=0.041 *
Second wave	−0.146 [−0.225, −0.067]<br>p<0.001 ***	
Third wave	−0.144 [−0.205, −0.084]<br>p<0.001 ***	
Policy stringency (lag 5) × Second wave	0.032 [0.017, 0.047]<br>p<0.001 ***	
Policy stringency (lag 5) × Third wave	0.034 [0.024, 0.044]<br>p<0.001 ***	
Flevoland (FL) [vs. Drenthe (DR)]	0.896 [0.006, 1.785]<br>p=0.048 *	1.449 [0.626, 2.273]<br>p<0.001 ***
Friesland (FR)	1.703 [1.115, 2.291]<br>p<0.001 ***	2.496 [1.898, 3.095]<br>p<0.001 ***
Gelderland (GE)	−0.195 [−0.439, 0.049] p=0.117	−0.141 [−0.370, 0.088]<br>p=0.228
Groningen (GR)	2.881 [1.662, 4.099]<br>p<0.001 ***	4.118 [2.816, 5.420]<br>p<0.001 ***
Limburg (LI)	0.987 [0.475, 1.499]<br>p<0.001 ***	1.409 [0.821, 1.997]<br>p<0.001 ***
Noord-Brabant (NB)	1.074 [0.599, 1.550]<br>p<0.001 ***	1.684 [1.217, 2.152]<br>p<0.001 ***
Noord-Holland (NH)	0.343 [−0.549, 1.234]<br>p=0.451	0.527 [−0.380, 1.433]<br>p=0.255
Overijsel (OV)	1.420 [0.897, 1.943]<br>p<0.001 ***	2.182 [1.646, 2.719]<br>p<0.001 ***
Utrecht (UT)	−1.353 [−2.024, −0.683]<br>p<0.001 ***	−1.742 [−2.362, −1.123]<br>p<0.001 ***
Zeeland (ZE)	0.008 [−0.330, 0.346]<br>p=0.963	−0.063 [−0.367, 0.241]<br>p=0.684
Zuid-Holland (ZH)	0.337 [−0.529, 1.202]<br>p=0.446	0.519 [−0.358, 1.396]<br>p=0.246
FL × Policy stringency (lag 5)		0.001 [−0.015, 0.016]<br>p=0.909
FR × Policy stringency (lag 5)		−0.007 [−0.022, 0.008]<br>p=0.371
GE × Policy stringency (lag 5)		−0.018 [−0.033, −0.002]<br>p=0.023 *
GR × Policy stringency (lag 5)		−0.009 [−0.024, 0.007]

	**Model S4a log(IRR)**	**Model S4b log(IRR)**
		p=0.264
LI × Policy stringency (lag 5)		−0.028 [−0.044, −0.012] p<0.001 ***
NB × Policy stringency (lag 5)		−0.024 [−0.040, −0.008] p=0.002 **
NH × Policy stringency (lag 5)		−0.011 [−0.027, 0.004] p=0.151
OV × Policy stringency (lag 5)		−0.014 [−0.029, 0.002] p=0.080 +
UT × Policy stringency (lag 5)		−0.015 [−0.030, 0.001] p=0.061 +
ZE × Policy stringency (lag 5)		0.003 [−0.013, 0.018] p=0.736
ZH × Policy stringency (lag 5)		−0.012 [−0.027, 0.004] p=0.135
Num.Obs.	*1596*	*1596*
R2 Adj.	*0.196*	*0.184*

*The numbers show the unstandardized coefficients from linear regression models, which indicate the implied change on excess mortality (defined as the log of IRR, or Incidence Rate Ratio) for a one-unit change in the covariate. The models include indicators at the province level (N=12), as well as controls for the demographic structure of the provinces (share of 65+, share of women, share of low-income households, share of 1st generation immigrants). 95% Confidence intervals are reported in the square brackets. Significance levels of p values: *** < 0.001; ** < 0.01; *<0.05; +<0.10. The precise p values when >0.01 are printed.*

***Table S5.* Number of registered COVID-19 deaths as a function of policy stringency, changes in mobility and additional covariates.**

	**Model S5a N Deaths**	**Model S5b N Deaths**	**Model S5c N Deaths**	**Model S5d N Deaths**	**Model S5e N Deaths**
Number of COVID-19 deaths (lag 1)	0.820 [0.795, 0.844] p<0.001 ***	0.818 [0.795, 0.842] p<0.001 ***	0.812 [0.788, 0.836] p<0.001 ***	0.822 [0.798, 0.846] p<0.001 ***	0.818 [0.795, 0.842] p<0.001 ***
Public holidays	0.980 [−1.505, 3.465] p=0.439	0.639 [−1.743, 3.022] p=0.599	1.125 [−1.219, 3.469] p=0.347	1.365 [−1.000, 3.731] p=0.258	0.929 [−1.407, 3.265] p=0.436
Minimum temperature	−0.589 [−0.716, −0.463] p<0.001 ***	−0.542 [−0.665, −0.418] p<0.001 ***	−0.622 [−0.749, −0.496] p<0.001 ***	−0.567 [−0.702, −0.433] p<0.001 ***	−0.647 [−0.774, −0.519] p<0.001 ***
Second wave	0.366 [−1.570, 2.303] p=0.711	0.609 [−1.295, 2.513] p=0.530	0.553 [−1.331, 2.436] p=0.565	1.002 [−0.905, 2.909] p=0.303	−1.492 [−3.513, 0.530] p=0.148
Third wave	−4.696 [−7.447, −1.946] p<0.001 ***	−3.467 [−6.178, −0.756] p=0.012 *	−4.428 [−7.158, −1.699] p=0.001 **	−3.741 [−6.640, −0.842] p=0.011 *	−6.345 [−9.195, −3.495] p<0.001 ***
Policy stringency index (lag 5)	−2.014 [−2.349, −1.679] p<0.001 ***	−1.428 [−1.791, −1.064] p<0.001 ***	−0.949 [−1.365, −0.534] p<0.001 ***	−1.682 [−2.022, −1.342] p<0.001 ***	−0.499 [−0.989, −0.009] p=0.046 *
Work (lag 6)		0.135 [0.072, 0.198] p<0.001 ***			
Transport (lag 6)			0.171 [0.116, 0.225] p<0.001 ***		
Groceries (lag 6)				0.060 [−0.015, 0.135] p=0.119	
Residence (lag 6)					−0.928 [−1.201, −0.654] p<0.001 ***
Num.Obs.	*1596*	*1578*	*1574*	*1582*	*1584*
R2 Adj.	*0.846*	*0.859*	*0.861*	*0.857*	*0.861*

*The numbers show the unstandardized coefficients from linear regression models, which indicate the implied change on the number of COVID-19 deaths for a one-unit change in the covariate. The models include indicators at the province level (N=12), as well as controls for the demographic structure of the provinces (share of 65+, share of women, share of low-income households, share of 1st generation immigrants). 95% Confidence intervals are reported in the square brackets. Significance levels of p values: *** < 0.001; ** < 0.01; *<0.05; +<0.10. The precise p values when >0.01 are printed.*

Fig S3 **Descriptive trends of the main variables of interest**

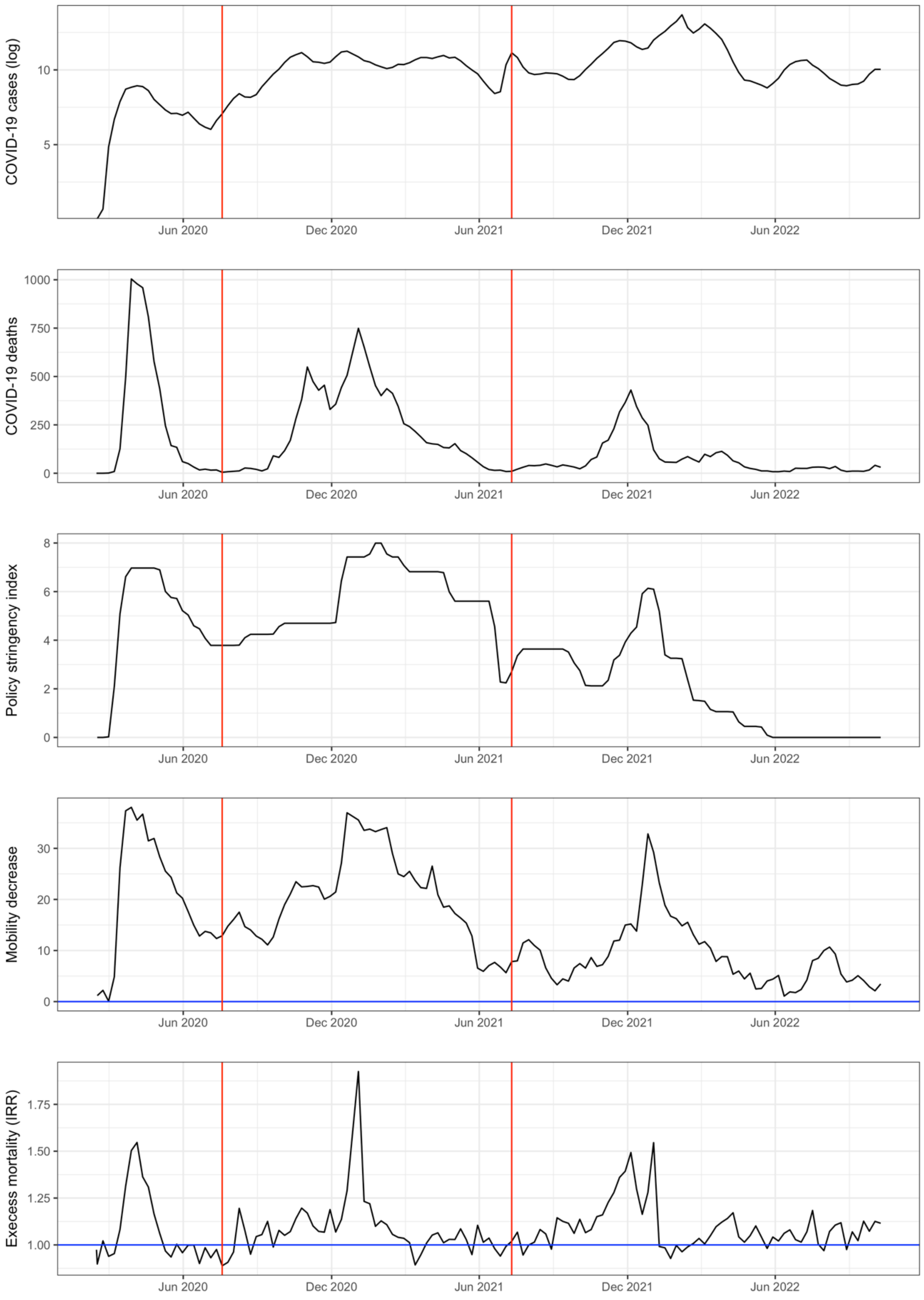

Note: Weekly data between February 2020 and October 2022 in The Netherlands. The vertical red lines show when the second and third COVID-19 waves started. From top to bottom: log of registered COVID-19 cases; number of COVID-19 deaths; Policy stringency index (scaled between 0 and 10); Mobility decrease (average of four indexes from the Google Mobility Report for The Netherlands as whole; the original values have been scaled so that higher values indicate less presence in public places and more presence at home); Excess mortality (log of the IRR, or Incidence Rate Ratio, for The Netherlands as a whole).

Fig S4 **Excess mortality per province**

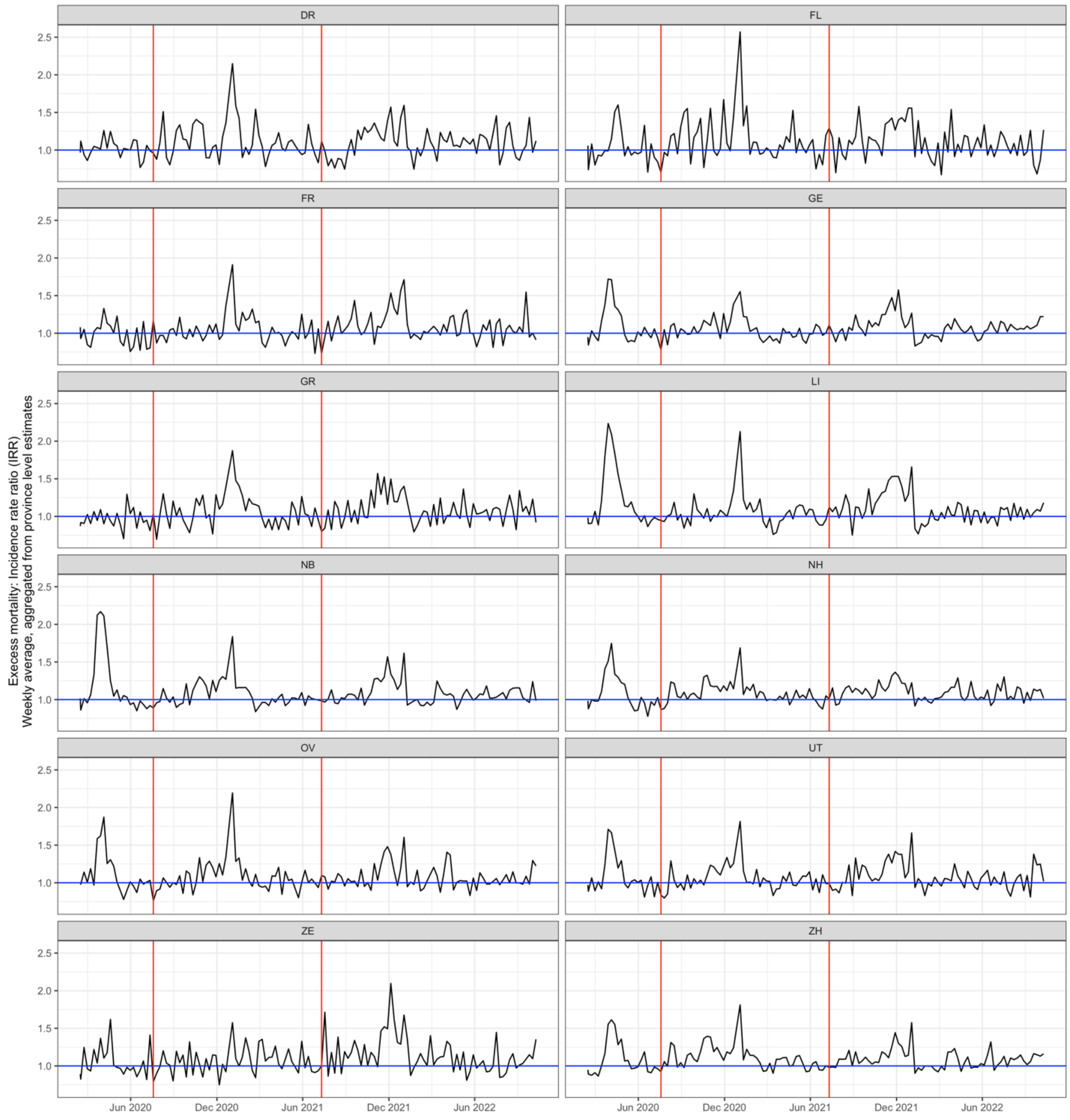

Note: Estimates of excess mortality (log IRR) in each of the twelve Dutch provinces. Weekly data between February 2020 and October 2022 in The Netherlands. The vertical red lines show when the second and third COVID-19 waves started. For province codes, see Table S3.

Table S6. Information about the provinces in The Netherlands

Name in Dutch	Name in English	Total population	Population density (per km²)
Drenthe	Drenthe	495,000	187
Flevoland	Flevoland	445,000	317
Friesland	Friesland	650,000	194
Gelderland	Gelderland	2,100,000	424
Groningen	Groningen	590,000	251
Limburg	Limburg	1,120,000	527
Noord-Brabant	North Brabant	2,600,000	523
Noord-Holland	North Holland	2,900,000	1,080
Overijssel	Overijssel	1,170,000	351
Utrecht	Utrecht	1,380,000	931
Zeeland	Zeeland	390,000	215
Zuid-Holland	South Holland	3,800,000	1,374
Nederland	The Netherlands	17,900,000	526

Note: Data for 2023 from the Central Statistics Bureau (CBS).

Table S7. Acknowledgements

This article is a result of a broader research project in which the following team participated:

Research group team (alphabetical order per institution):

Name	Affiliation	ORCID
Dr. A van Hylckama Vlieg	1	0000-0001-7386-1168
Dr. C Caram-Deelder	1	0000-0003-3161-5684
Prof. dr FR Rosendaal	1	0000-0003-2558-7496
Dr. Q Chen	1	0000-0001-9669-0007
Prof. dr. RHH Groenwold	1,2	0000-0001-9238-6999
Dr. DO Mook-Kanamori	1,3	0000-0002-0182-5122
BSc F Tari	1,3	0009-0007-2601-0476
Prof. dr. SC Cannegieter	1,4	0000-0003-4707-2303
Prof. dr. OM Dekkers	1,5	0000-0002-1333-7580
Dr. ML Haaksma	3	0000-0002-3518-9152
MD. EAS Koster	2	0000-0002-8446-5133
Dr. LC de Wreede	2	0000-0002-7667-9369
MSc. MH Sluiskes	2	0000-0002-2063-3492
Dr. BJ Carroll	6	0000-0002-9466-315X
Dr. DD Toshkov	6	0000-0002-7444-9340
Prof. dr. K Yesilkagit	6	0000-0001-9660-7859
Dr. C van Nieuwkoop	3,7	0000-0003-0734-0844
Dr. M van Aken	3,7	0000-0002-6686-5459

1 Department of Clinical Epidemiology, Leiden University Medical Center, Leiden, The Netherlands
2 Department of Biomedical Data Sciences, Leiden University Medical Center, Leiden, The Netherlands
3 Department of Public Health and Primary Care, Leiden University Medical Center, Leiden, The Netherlands
4 Department of Internal Medicine Section of Thrombosis and Haemostasias, Leiden University Medical Center, Leiden, The Netherlands
5 Departments of Endocrinology and Metabolism, Leiden University Medical Center, Leiden, The Netherlands
6 Faculty of Governance and Global Affairs, Leiden University, Leiden, The Netherlands
7 Haga Hospital, The Hague, The Netherlands
