# Supplementary material for: The impact of policy restrictions and mobility changes on excess mortality during the COVID-19 pandemic in The Netherlands, 2020–2022
Source: PLoS One. 2026 Feb 9;21(2):e0322350. doi: 10.1371/journal.pone.0322350 (PMC12885301; doi:10.1371/journal.pone.0322350)
Supplement: Attachment — Submitted filename: PLOS_ONE_peer_review.pdf [file pone.0322350.s003.pdf]

Review of **The Impact of Policy Restrictions and Mobility Changes on Excess Mortality During the COVID-19 Pandemic in The Netherlands, 2020-2022**

PLOS ONE

May 12, 2025

1 Summary

The study's merit lies in its ability to provide concrete estimations of the size and impacts of stringency policies during three waves of COVID-19 pandemics. For that, authors rely on panel data, with observations from different regions within a single country. In my interpretation, if this is done in a compelling manner, according to reviewers' guidelines, the paper would be suitable for publication in PLOS ONE, as an original research, given the current state of literature, far from reaching a final conclusion on the topic.

What remains to be assessed, therefore, is the soundness of the identification strategy used. At this point, I am concerned the paper, in the current form, is not suitable for publication, as many parameters and assumptions are made throughout the text, and left unexplained, at the expense of a compelling argument about the plausibility of the causal claims.

2 Causal claims

The main claims made by the authors are as follows:

- COVID-19 impacts policy decisions, differently across waves. This is estimated in Table 1, showing, for example, that one additional death in the previous week **causes** policy makers to increase the stringency index in 0.01 (from 0 to 10). Also, doubling the cases (divided by 1.000) induces a change of 0.35 in the stringency index. **This information is not used anymore in the identification strategy.**
- Impacts on mobility are estimated in Table 2, with both COVID-19 and policy decisions as independent variables. A causal claim is made that changing one unit within the range (0-10) in stringency, translates into around 3 p.p. in work and transport mobility, from baseline. It is clear that severity of COVID-19 is a confounding factor impacting both policy changes and mobility. **The question is: using COVID-19 deaths and cases (with a 1 week lag) as a covariate cleans the threats to causal estimation of the impact of policy on mobility?**
- Impacts of mobility **and** policy changes are estimated in Tables 3 (cases) and 4 (deaths), using lags of 2 weeks for cases and 6 weeks for deaths in the regressors. Authors use these results, together with the results of Table 2 to make the final causal claims of policy changes impacting COVID-19 deaths and cases **both directly and through mobility changes**.
- Below I present a causality diagram pointing to the tables where results are presented.

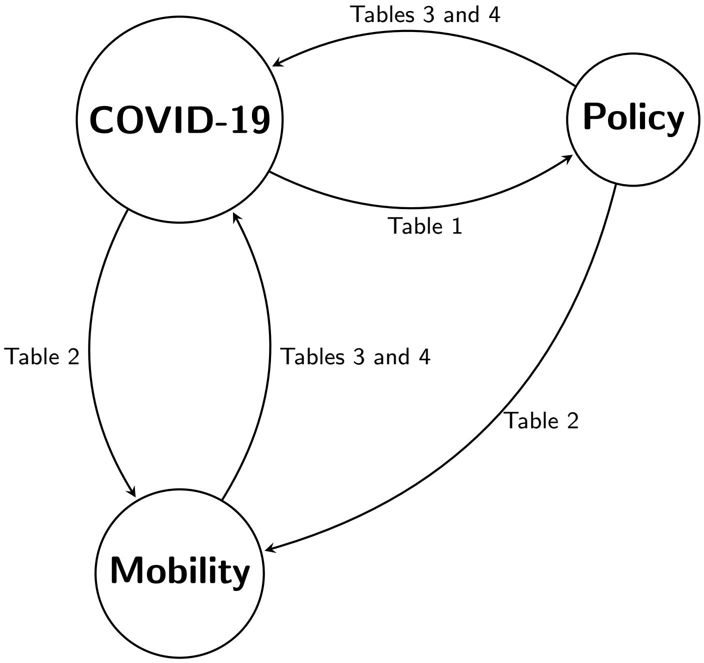

It is my interpretation that the paper should make a **more compelling argument on the necessary conditions for causality and how they are met, relying on the existing literature**, to convince readers that the strategy is as clean as possible and overcomes any possible threats, **specially endogeneity coming from this circular causality framework**. I am not completely aware of the literature's state of the art on this instance, but the authors should elaborate on how considering the lags of variables solves the problem, and how to put together the three different regression results to make one compelling argument.

Also, I am very concerned about the excess use of parameters and assumptions at the author's discretion with no proper justification or robustness tests. Examples are: (i) rescaling of the stringency index; (ii) the definition of 'Information' variable using log(cases/1.000) on one hand, and new COVID-19 deaths in level (and lagged) at the other; (iii) the definition of what Google Mobility variables enter the study; (iv) definitions of lags being 1 week at first, then 6 weeks, then 5 weeks, all in the main specifications.

Finally, for the first regression, presented in Table 1, besides the fact that it is actually not used in the study's argument, I have another comment: it is perhaps not clear, but it is my understanding that any observations $stringency_{i,t}$ and $stringency_{j,t}$ are exactly the same for any pair (i, j) of provinces, as stringency is measured at the country level. If this is the case, I am not sure the regression makes any sense at all. In fact, I'd assume policy makers evaluate cases and death numbers at the national level (i.e. simultaneously assessing all provinces' outcomes) to define a nation-wide policy. What type of causal claim can be made here with that premise in mind and considering there is no variation at the province level?

3 Full comments throughout the text

- Line 16: it is a system, but there is no actual structure considered between results of the three different regressions to construct one single identification strategy. I'd remove the word here to avoid misleading readers into understanding it is a structured approach.
- Line 58: "This is because the number of deaths (...)" is a circular reasoning. I believe the argument for using excess mortality should be explored further.
- Line 62: "while" is a confusing conjunction in this sentence.

- Line 66: "dynamic of the pandemic" is referring to the waves, I believe. should be made clear. Also, the definition of waves dummies is not clear in any part of the text, as I recall, this should be done appropriately.
- Line 67: "One of our assumptions" is actually not an assumption, it is something being tested by the proposed framework.
- Line 82: variation is needed to make causal claims, of course. The question here would be on whether increased stringency is causing reduced deaths or there is a third factor impacting both. It is not straightforward that this variation allows causal estimation. Moreover, has the impacts of vaccines been considered in the research design? Isn't it a threat to identification? I believe it should be explored over the results, maybe citing when evaluating impacts of different waves. A robustness test with only the pre-vaccine period would provide good insight perhaps.
- Line 120: "area" should be "are".
- Line 155: I highly recommend changing the system of equations to a causal diagram (like the one I presented before). Besides helping in interpretation, IMHO using equations here directly points to a structural approach, or at least an identification strategy where results from one regression is used to tackle the threats to causal claims in the others (two or three stages approach). This is not the case, and should be made very clear to the reader. In the diagram, the lags used should be added; and I believe this is a good part of the text to explore much more on (i) the decision process for the lags to be used; and (ii) how using lags completely cleans the identification strategy, according to literature.
- Line 167: wouldn't all regressions benefit from fixed effects for the provinces in terms of causal interpretation? Maybe this is a path to explore.
- Line 171: it should be clearer that the policy stringency index is the same for all provinces. Right?
- Line 173: explain the rescaling decision.
- Lines 174-177: explain the functional forms used. Why log(cases/1.000) and lag number of deaths?
- Line 185: why the categories "parks", and "retail and recreation" are ignored?
- Line 202: tested any other holidays? King's day?
- Lines 221-222: why the different treatment for cases and deaths?
- Footnote 5: have you tested Poisson regression or the inverse hyperbolic sin approaches? Would be good to present as robustness tests.
- Table 1: what "exact p values" mean in this context?
- Line 228: see my comment in the previous section of this review about the validity of the first regression, if there is no variation in policy data at province level.
- Table 2: we should remember that results for residential mobility variable are different from the others, as they measure changes in time spent at home, and not number of visitors.
- Table 2: differently from the first regression, now log is used for both deaths and cases (with a lag 1). Discretion and choices of parameters must be explained and robustness tests are good practice.
- Line 272: "maximum average temperature" can mean different things, should be explained better. Is it the maximum of daily average? Or is it a dummy for being the week with highest average temperature? In the variables description section nothing is said about a maximum operator.
- Line 277: "but remain significant and in the direction reported in Table 2", unless there are typos, or I am misreading, this is not true from what is actually presented in Table 2.

- Line 278: Table S1 should be in the text, specially if you are drawing conclusions from it, as follows. I suggest adding the models with interactions in Table 2, as it was done for Table 1.
- Line 293: change "fixed effects" for "dummies" or "indicators" in this context.
- Line 294: explain the use of maximum operator.
- Table 3: results from holidays make sense, I just have one comment, should we something be said about gatherings in holiday events? Can residential presence mean actually families are gathering, from being alone, which could increase cases and deaths?
- Line 334: now lag is 6 weeks, how is this choice done?
- Line 340: I believe the reference is for "Model 4b".
- Line 347: If the lag matters, we should see a full sensibility test and an explanation for the choice done.
- Lines 360-362: Makes sense, you may have a flat (high) level of COVID cases, and this compromises hospital capacity to handle higher holiday related deaths (e.g. road accidents, alcohol related). I'd explore this argument in the text.
- Lines 365-367: the heterogeneous effects correlating with density is a compelling result, can you explore further to mobility effects?
- Line 371: "largely" should be "large".
- Line 373: why just from 4 to 7 in the sensibility analysis? 1,2,3 don't matter?
- Line 377: why fix at 5? isn't 6 the preferred specification lag?
- Line 379: this is what worries me about the whole identification strategy. It seems unclean in many instances, in the sense that discretion is used for a lot of parameters, and many assumptions are present. Causal claims are much less compelling if this is done. For the sake of the argument: should we make the causal claim that decreasing mobility in transportation increases deaths after 4 weeks? Or time spent at home?
- Line 393: are the results robust to fixed effects test the ones in Table S3 from supporting information?
- Lines 413-415: reading the whole text and results tables, I was under the impression that stringency alone (and not through mobility changes) was the main driver of cases and deaths reduction (see, for example, lines 333-342; 110-112). The conclusion here leads the reader to believe it is rather a very clear path from stringency, to mobility change, to reduction in deaths and cases. You should review, or make results presentation more clear, in case I misunderstood the general impacts.
- Lines 424-425: grocery stores and pharmacies are one category and retail and recreation are another in Google Mobility data, right? This should be reviewed, as in the data presentation it said grocery is used, but result tables present as "retail", and here both are cited. What is actually being used? And why?
- Lines 428-430: Again, I was under the impression that policy induced mobility changes was the "marginal" effect in the findings, and not the main channel.
