# Supplementary material for: The impact of policy restrictions and mobility changes on excess mortality during the COVID-19 pandemic in The Netherlands, 2020–2022
Source: PLoS One. 2026 Feb 9;21(2):e0322350. doi: 10.1371/journal.pone.0322350 (PMC12885301; doi:10.1371/journal.pone.0322350)
Supplement: Attachment — Submitted filename: PLOS_ONE_peer_review__round_2_-2.pdf [file pone.0322350.s005.pdf]

Review of **The Impact of Policy Restrictions and Mobility Changes on Excess Mortality During the COVID-19 Pandemic in The Netherlands, 2020-2022**

PLOS ONE

August 23, 2025

1 Review

Reading the new version and the authors' answers, I find they did mostly a good job on addressing my concerns, but with no significant methodological improvements. What the authors did was to make clear throughout the text the majority of their modeling choices and possible threats to identification, still not precisely explaining them, except for the further elaboration on the direct answers to the review, which are mostly satisfactory.

As a final assessment, it is my understanding that the paper has 3 main threats to validity, that are now fully transparent and clearly explained by the authors in the conclusion and empirical approach sections. I outline them below in my terms, and leave for the Editor the judgment if it's fit for the journal with these possible gaps in mind.

- No cross-sectional variance of treatment harms causal interpretation of results, mainly in the first regression.
- No structural approach to deal with the results of the three stages to make one compelling causal argument, with bias correction.
- The assumption that treatment assignment is as good as random conditional on internally measured cases and other confounders is now very clear in the text, but is usually considered too strong in settings like this.

If the decision is to accept publication, these are the minor revisions I'd still recommend. Mostly small typos.

- Line 180: should be: "readability of our results"
- Lines 211-213: is there a reason to consider cases impacted by the temperature and deaths by the peaks? Instead of the illustrative example on the subsequent lines, I'd take the chance to explain this different treatment.
- Line 229: should be "presents five linear regressions".
- Line 353: should be "This implies".

Also, I understand the challenges with word counting, but I'd push for further adding some of the richer explanations given as direct answers to my comments in the text, focusing on being concise on other, less critical, parts of the presentation.
