# Supplementary material for: The impact of policy restrictions and mobility changes on excess mortality during the COVID-19 pandemic in The Netherlands, 2020–2022
Source: PLoS One. 2026 Feb 9;21(2):e0322350. doi: 10.1371/journal.pone.0322350 (PMC12885301; doi:10.1371/journal.pone.0322350)
Supplement: Attachment — Submitted filename: Rebuttal letter 2.pdf [file pone.0322350.s006.pdf]

Dear Editor,
Thank you for the invitation to submit a revised version of the manuscript.

We are happy that the manuscript is considered sharply improved after the first-round revisions. It is also reassuring that Reviewer 2 considers that the main threats to validity are now ‘fully transparent and clearly explained’.

We understand the remaining concerns about the limitations of the analyses with regard to endogeneity.

As suggested by the Editor, we have tried again to make it even clearer that this limitation is important, and we have edited the interpretation of our results with regard to causality, in light of this concern. Our detailed responses are outlined below.

Reviewer 2
Thank you for the clear statement of the main assumptions relevant for the limitations of our paper. Please see the new sentence added to the introduction (also pasted below in response to Reviewer 2) that makes it even clearer that our work does not provide a definitive causal identification of the policy effects, but it still delivers relevant information.

We also added the following sentence to the conclusion:

“While we cannot rule out all sources of confounding due to the endogenous nature of policy adoption, our conditional association framework provides valuable evidence for policy deliberation.”

Thank you also for the identified typos and other small issues, which now have been corrected.

Reviewer 3
In response to the Reviewer’s concern, we replaced most mentions of ‘policy effects’ with ‘policy-related reductions’, and we removed some other instances of causal language (e.g. ‘led to’).

We have also added the following sentence to the introduction: ‘While endogenous policy adoption precludes definitive causal identification, we provide policy-relevant evidence by estimating conditional associations that control for the pandemic information available to decision-makers. Specifically, we address what mortality reductions accompanied policy restrictions given the conditions that prompted them, that is, the practical question facing policymakers during the pandemic.’

The threat of reverse causality between cases/deaths and policy adoption is certainly not eliminated in our analysis, but the measures we take provide a reasonable safeguard against this possibility. The temporal dimension of our data is relatively fine-grained, and we control for different lags (and explore whether the precise definition of the lag structure makes a difference). Yet, we do not disagree that a risk of bias remains, and we are clear about this limitation of our approach. Still, compared to the state-of-the-art, our approach provides an improvement by using excess mortality as an

outcome variable, combining mobility and policy data, and using region-specific estimates of all major variables.

The bridge between the empirical conditional correlations that we present and causal claims is always reliant on assumptions (following the causal framework of Judea Pearl). This is true for all research designs, but is especially pertinent for designs based on observational data, which are the only ones available when it comes to estimating the effects of COVID-19 related policy interventions. The best that we can do is be clear about the assumptions that we make (which we believe is already the case), try different assumptions (which we do by varying the lag structure) and relax some of the assumptions when possible (which would be the case if we had credible instruments, for example).
