# Supplementary material for: The impact of policy restrictions and mobility changes on excess mortality during the COVID-19 pandemic in The Netherlands, 2020–2022
Source: PLoS One. 2026 Feb 9;21(2):e0322350. doi: 10.1371/journal.pone.0322350 (PMC12885301; doi:10.1371/journal.pone.0322350)
Supplement: Attachment — Submitted filename: responses to reviewers.docx [file pone.0322350.s004.docx]

Dear Editors,

Thank you very much for the opportunity to revise our paper and the helpful and constructive comments of the reviewers. We have revised the draft manuscript in response to the comments. Most importantly, we explain our causal estimation strategy more clearly, and we provide additional checks for the robustness of our main results. We believe the manuscript is now clearer and stronger and would make a suitable contribution to PLOS ONE. A detailed overview of our responses is included below:

# Journal Requirements

Response:

(1) We have adjusted the formatting of the text and file naming in line with the journal requirements.

(2) The funding information has been removed from the Acknowledgements Section. The funding information in the Funding Statement can remain as it is.

(3) As indicated, we will make all data and coded necessary for reproducing the results of the paper openly available via the Harvard Dataverse, upon acceptance of the paper.

(4) We have kept the SI as a complete file to ease the review process. We will reformat the file in line with the journal requirements upon acceptance.

# Academic Editor

Response: In the following table we respond to the comments that the ScreenIT Report flagged as ‘review for potential issues’ (the report did not flag any critical issues).

| **Comment** | **Response** |
| --- | --- |
| We did not find a study flow chart of excluded observations. We strongly recommend using flow charts because they provide an overview of the study design and more information about attrition. If you included a study flow chart in your supplemental files, we apologize for missing this. Our tool is not able to screen separate supplemental files. | We have not excluded any observations. |
| Not detected. If you performed an experimental study, please specify whether participants, animals or samples were randomly assigned to treatments or groups, and specify the randomization procedure. | This is not an experimental study so there is no randomization. |
| Not detected. Please specify whether blinding was used at various phases of the experiment (e.g., blinding of patients, caregivers, outcome assessments, data analysis). | This is not an experimental study so there is no blinding. |
| Not detected. Please state how you determined the sample size for the study. If you performed a power or sample size calculation, please provide details. | The sample size includes all observations (week-province) for which data is available. |
| We found one or more references to open code in your manuscript that did not seem to be included in your code availability statement: "".If these are references to your own code, make sure to include them in the code availability statement. If these are code citations, make sure to clearly state that you used previously published code. | We will make the scripts in R necessary for replication of our analyses publicly available upon acceptance. References to code contained in specific R packages used in the analyses is already included in the ‘Materials and methods’ section. |
| If permitted, sharing data on a public repository with a persistent identifier (a DOI or accession number) can improve reproducibility and make it easier for other scientists to expand on your work. Papers with open data are cited more often than papers without open data. Some institutions have an expert who can provide advice on data sharing. | As already indicated, we will make replication data available on a public repository. |
| An explicit section about the limitations of this study was not found. We encourage authors to include a paragraph in the discussion that addresses study limitations. Every study has limitations. Describing these limitations helps readers to understand and contextualize the research. | We have now included a short paragraph outlining the main limitations of our research. |
| It is important to supply a conflict of interest statement, even if there are none to declare, to transparently present the research. | We now include a disclosure statement. |
| It is important to supply a funding statement  because it makes the funding source and  potential conflicts of interests transparent. | A funding statement has been submitted via the online form for the manuscript submission. |

# Reviewer 2

*The study's merit lies in its ability to provide concrete causal estimations of the size and impacts of stringency policies during three waves of COVID-19 pandemics. For that, authors rely on panel data, with observations from different regions within a single country. In my interpretation, if this is done in a compelling manner, according to reviewers' guidelines, the paper would be suitable for publication in PLOS ONE, as an original research, given the current state of literature, far from reaching a final conclusion on the topic.

(1) What remains to be assessed, therefore, is the soundness of the identification strategy used. At this point, I am concerned the paper, in the current form, is not suitable for publication, as many parameters and assumptions are made throughout the text, and left unexplained, at the expense of a compelling argument about the plausibility of the causal claims.*

Response: We have removed some modelling choices that could have caused confusion (e.g. scaling). We are more consistent in defining the lag structure. Most importantly, we have conducted numerous additional robustness analyses that show that our main results are not sensitive to many of the secondary assumptions about variable transformations, lags, etc.

*(2) It is my interpretation that the paper should make a more compelling argument on the necessary conditions for causality and how they are met, relying on the existing literature, to convince readers that the strategy is as clean as possible and overcomes any possible threats, specially endogeneity coming from this circular causality framework. I am not completely aware of the literature's state of the art on this instance, but the authors should elaborate on how considering the lags of variables solves the problem, and how to put together the three different regression results to make one compelling argument.*

Response: we agree that the assumptions for the causal claims made in the paper are crucial. We exaplin now more clearly, that the causal identification strategy hinges on the assumptions that once the information about the dynamic of the pandemic is taken into account (in terms of number of cases and deaths and their growth rates), the changes in the policy index can be considered exogenous. In this approach, we follow existing literature (e.g. Chernozhuhov et al. 2021), although their approach is much more complex, as they have cross-sectional variation in the timing of policy imposition but a much shorter time-period of analysis and because we do not aim to estimate the cumulative effects of the restrictive policies but only one-step ahead effects.

*(3) Also, I am very concerned about the excess use of parameters and assumptions at the author's discretion with no proper justification or robustness tests. Examples are: (i) rescaling of the stringency index; (ii) the definition of 'Information' variable using log(cases/1.000) on one hand, and new COVID-19 deaths in level (and lagged) at the other; (iii) the definition of what Google Mobility variables enter the study; (iv) definitions of lags being 1 week at first, then 6 weeks, then 5 weeks, all in the main specifications.*

Response: please see the general response above and the specific responses in the table below

*Finally, for the first regression, presented in Table 1, besides the fact that it is actually not used in the study's argument, I have another comment: it is perhaps not clear, but it is my understanding that any observations $stringency_{{i,t}}$ and $stringency_{j,t}$ are exactly the same for any pair $(i,j)$ of provinces, as stringency is measured at the country level. If this is the case, I am not sure the regression makes any sense at all. In fact, I'd assume policy makers evaluate cases and death numbers at the national level (i.e. simultaneously assessing all provinces' outcomes) to define a nation-wide policy. What type of causal claim can be made here with that premise in mind and considering there is no variation at the province level?*

*3 Full comments throughout the text*

Response: We are grateful to the reviewer both for the general comments and the list of specific points identified below. This has been very helpful in identifying the areas where we could explain better and improve our analyses and presentation. We list the specific responses in the table below.

| **Comment** | **Response** |
| --- | --- |
| **Introduction & Theoretical causal mechanisms** |  |
| • Line 16: it is a system, but there is no actual structure considered between results of the three different regressions to construct one single identification strategy. I’d remove the word here to avoid misleading readers into understanding it is a structured approach. | We removed “system” from the sentence. |
| • Line 58: ”This is because the number of deaths (...)” is a circular reasoning. I believe the argument for using excess mortality should be explored further. | To this statement we have added brief reasoning why excess mortality should be used in place of registered deaths. |
| • Line 62: ”while” is a confusing conjunction in this sentence. 2 | We replaced “while” with “and”. |
| • Line 66: ”dynamic of the pandemic” is referring to the waves, I believe. should be made clear. Also, the definition of waves dummies is not clear in any part of the text, as I recall, this should be done appropriately. | We replaced ‘dynamic of the pandemic’ with ‘changes in registered cases and differences between successive pandemic waves’ to clarify that both form the ‘dynamic’ component. We also add a sentence in the description of ‘additional covariates’ that defines the wave cut-offs. |
| • Line 67: ”One of our assumptions” is actually not an assumption, it is something being tested by the proposed framework. | We have rephrased this sentence to avoid “assumption” language. |
| • Line 82: variation is needed to make causal claims, of course. The question here would be on whether increased stringency is causing reduced deaths or there is a third factor impacting both. It is not straightforward that this variation allows causal estimation. Moreover, has the impacts of vaccines been considered in the research design? Isn’t it a threat to identification? I believe it should be explored over the results, maybe citing when evaluating impacts of different waves. A robustness test with only the pre-vaccine period would provide good insight perhaps. | As any other causal identification approach, ours also hinges on important assumptions. The most important one – as we now note in the text – is that changes in policy can be considered exogenous (‘as random’) conditional on the included covariates, and those capturing the information about the state of the pandemic in particular. We are less worried about a confounder of the imposing of policy restrictions and excess mortality than about dynamic, reciprocal effects between cases and policies. But following existing econometric approaches, we let the lag structure of the relationships address this concern.  About the vaccinations: mass vaccinations became available in The Netherlands during the course of 2021 and only reached the majority of the population in the second part of the year. Therefore, only the analysis of the third wave is possibly affected by the availability of vaccination. The smaller policy effects that we note during the third wave could be due to the progress of the vaccination campaign. We have added a sentence to the conclusion mentioning this possibility. |
| • Line 120: ”area” should be ”are”. | This is fixed. |
| • Line 155: I highly recommend changing the system of equations to a causal diagram (like the one I presented before). Besides helping in interpretation, IMHO using equations here directly points to a structural approach, or at least an identification strategy where results from one regression is used to tackle the threats to causal claims in the others (two or three stages approach). This is not the case, and should be made very clear to the reader. In the diagram, the lags used should be added; and I believe this is a good part of the text to explore much more on (i) the decision process for the lags to be used; and (ii) how using lags completely cleans the identification strategy, according to literature. | We do not indeed estimate a system of structural equations, and we have removed references that might suggest so. We also moved the first part of the analysis to the SI for this purpose. We estimate the equations independently.  Whether we do or not we should clarify that we do not estimate a system of structural equations, as reviewer 2 thinks our approach could be misconstrued as such and reviewer 3 may think we actually do. |
| **Materials and methods** |  |
| • Line 167: wouldn’t all regressions benefit from fixed effects for the provinces in terms of causal interpretation? Maybe this is a path to explore. | We include Indicators (fixed effects) for provinces in all regressions except those in which policy stringency and changes in it are the outcome. We do not in these because these outcomes do not differ among provinces. |
| • Line 171: it should be clearer that the policy stringency index is the same for all provinces. Right? | We now note this in the description for this variable. |
| • Line 173: explain the rescaling decision. | We briefly note the readability justification for this. In short, we do that to ease interpretation so that a one-unit change in the index corresponds to realistic policy interventions. |
| • Lines 174-177: explain the functional forms used. Why log(cases/1.000) and lag number of deaths? | Dividing the number of cases by 1,000 was initially considered in order to make the coefficient of this variable comparable to the rest, but the scaling turned to be unnecessary and we drop it from the current version of the paper to avoid confusion.  Applying the log function is justified because it is not reasonable to expect that the effects of this variable are on a linear scale, e.g. an increase of 100 cases has the same effect when the base level is 100 cases compared to when it is 10,000 cases.  At the same time, the effects of deaths could well be on a linear scale, given the scale of variation of this variable over time. In the equations modelling policy stringency, logging the number of deaths does not change the inference for the effect of the number of deaths.  The number of deaths is lagged in the models of policy stringency because it took more time for this measure to become available to policy-makers: while number of cases where reported daily, deaths were aggregated from different sources (individual hospitals and other places), which took time. |
| • Line 185: why the categories ”parks”, and ”retail and recreation” are ignored? | We exclude the category ‘Parks’ because there is no clear theoretical expectation about the effects of pandemic information and policy on presence in this category of places. Policy restrictions could have actually increased presence in ‘Parks’ as they provided places for people to be outside in relative isolation from other people. But some municipalities discouraged the use of public parks, while others did not have a clear policy.  **We added results concerning the category ‘Retail and recreation’ to all models.** |
| • Line 202: tested any other holidays? King’s day? | No other holidays have been tested. |
| **Results** |  |
| • Lines 221-222: why the different treatment for cases and deaths? | Information about the number of COVID-19 related deaths took a longer time to compile and be made available publicly and for decision-making purposes. |
| • Footnote 5: have you tested Poisson regression or the inverse hyperbolic sin approaches? Would be good to present as robustness tests. | The policy restrictions variable is continuous, which means that it has fractional values which are not expected under a Poisson distribution.  We applied the inverse hyperbolic sin transformation to the number of COVID-19 case (instead of log(x+1)). The results are substantively the same as the ones reported in the SI. |
| • Table 1: what ”exact p values” mean in this context? | We meant by that that we have printed the precise p value rather than referring to its value by a symbol. We have now changed the phrasing to make this clearer: ‘*The precise p values are printed when >0.01.’* |
| • Line 228: see my comment in the previous section of this review about the validity of the first regression, if there is no variation in policy data at province level. | There is indeed no variation in the policy stringency at the province level. We replicated the models using country-level data only, and substantively the results remain the same as the ones using province-level data with fixed effects and clustered standard errors. |
| • Table 2: we should remember that results for residential mobility variable are different from the others, as they measure changes in time spent at home, and not number of visitors. | Indeed, we have now separated this result in a separate sentence to make this clearer. |
| • Table 2: differently from the first regression, now log is used for both deaths and cases (with a lag 1). Discretion and choices of parameters must be explained and robustness tests are good practice. | We explained above why the number of deaths has remained untransformed in the models of policy stringency and noted that the effect of this variable is not sensitive to the decision whether to apply the log transformation or not.  In the case of mobility, it is reasonable to expect that people (but not governments!) get de-sensitised to the reported number of deaths once high levels are already reached, so that we log both the cases and the deaths.  Importantly, this transformation has no effect on the substantive results – entering the number of deaths untransformed in the models of mobility has no effect on the inference about the effect of policy stringency nor on the effects of COVID-19 cases or deaths.  We also re-estimated the model with the number of deaths lagged with two weeks instead of one – the conclusions remain the same. |
| • Line 272: ”maximum average temperature” can mean different things, should be explained better. Is it the maximum of daily average? Or is it a dummy for being the week with highest average temperature? In the variables description section nothing is said about a maximum operator. | We have now included a small paragraph clarifying how the measures of temperature are defined and used in the regression models. |
| • Line 277: ”but remain significant and in the direction reported in Table 2”, unless there are typos, or I am misreading, this is not true from what is actually presented in Table 2. 3 | Table S1 shows that there are significant interactions between the effect of policy stringency and the pandemic waves, so that the effect of policy stringency gets significantly smaller. The effect remains negative and significant, however. To illustrate this better, we include the new figure S2 in the SI. |
| • Line 278: Table S1 should be in the text, specially if you are drawing conclusions from it, as follows. I suggest adding the models with interactions in Table 2, as it was done for Table 1. | We are facing limitations in terms of word limit for this article, which is why we keep the longer tables in the SI, but if the Editors also find this suggestion useful, we can include this table in the main text. |
| • Line 293: change ”fixed effects” for ”dummies” or ”indicators” in this context. | References to ‘fixed effects’ have been replaced with ‘indicators’ throughout the text. |
| • Line 294: explain the use of maximum operator. | We have clarified the definition of the variables and the use of the maximum operator. |
| • Table 3: results from holidays make sense, I just have one comment, should we something be said about gatherings in holiday events? Can residential presence mean actually families are gathering, from being alone, which could increase cases and deaths? | This is an interesting point. According to the documentation, however, the Residential category of the Google Mobility reports measures change in the *duration* of presence (unlike the other categories which track changes in the number of visitors). Presumably, large gathering in Residential places then would not have a big impact on the measure of duration. |
| • Line 334: now lag is 6 weeks, how is this choice done? | This was a typo. Policy has always been entered with lag 5. We have corrected the information. |
| • Line 340: I believe the reference is for ”Model 4b”. | Fixed. |
| • Line 347: If the lag matters, we should see a full sensibility test and an explanation for the choice done. | We report sensitivity analysis in Fig 4. The issue is that the contemporarious correlations between policy and mobility make it hard to estimate precisely the effects of both variables when they are entered at the same time in the models of excess mortality. That’s why Fig 4 shows the effects of mobility when the policy measure is excluded. All effects are then significant and in the expected direction, and are not too sensitive to the exact lag used. We have adjusted the text to reflect these results. |
| • Lines 360-362: Makes sense, you may have a flat (high) level of COVID cases, and this compromises hospital capacity to handle higher holiday related deaths (e.g. road accidents, alcohol related). I’d explore this argument in the text. | This possible interpretation has been added. |
| • Line 371: ”largely” should be ”large”. | Fixed. |
| • Line 373: why just from 4 to 7 in the sensibility analysis? 1,2,3 don’t matter? | When it comes to COVID-19 related deaths, lags lower than 4 weeks are unreasonable because of the time it takes for the disease to kill the person. |
| • Line 377: why fix at 5? isn’t 6 the preferred specification lag? | Again, apologies for the confusion. The lag for policy has always been 5 weeks. |
| • Line 379: this is what worries me about the whole identification strategy. It seems unclean in many instances, in the sense that discretion is used for a lot of parameters, and many assumptions are present. Causal claims are much less compelling if this is done. For the sake of the argument: should we make the causal claim that decreasing mobility in transportation increases deaths after 4 weeks? Or time spent at home? | We can see where the concern of the reviewer comes from, and we have tried to be more principled in these decisions (transformations, lags) in the current version of the paper. We also conducted numerous robustness analyses that show that these decisions do not impact the main conclusions.  With regard to the particular point mentioned here: The problem with the interpretation arises because of collinearity between the policy and mobility values. When the mobility aspects are entered without policy, their associations are consistent, precisely estimated and in the expected directions (if reductions in mobility decreased excess mortality). It is only when both policy and mobility are in the model that the effect of mobility is sensitive to the exact lag.  Hopefully the new version of Fig 3 makes this clear. |
| • Line 393: are the results robust to fixed effects test the ones in Table S3 from supporting information? | ??? |
| **Discussion and Conclusion** |  |
| • Lines 413-415: reading the whole text and results tables, I was under the impression that stringency alone (and not through mobility changes) was the main driver of cases and deaths reduction (see, for example, lines 333-342; 110-112). The conclusion here leads the reader to believe it is rather a very clear path from stringency, to mobility change, to reduction in deaths and cases. You should review, or make results presentation more clear, in case I misunderstood the general impacts. | This is the correct. We have changed the wording of the conclusion to reflect the fact that there is a significant effect of the policy measures that does not pass through mobility changes. |
| • Lines 424-425: grocery stores and pharmacies are one category and retail and recreation are another in Google Mobility data, right? This should be reviewed, as in the data presentation it said grocery is used, but result tables present as ”retail”, and here both are cited. What is actually being used? And why? | Thank you for noticing this source of potential confusion to the readers. We corrected the use. Since we also added the ‘Retail and recreation’ category to all analyses, we refer to it in short as ‘retail’, and to the other category as ‘groceries’. |
| • Lines 428-430: Again, I was under the impression that policy induced mobility changes was the ”marginal” effect in the findings, and not the main channel. | See above, we adjusted the wording to reflect better the empirical results. |

# Reviewer 3

*• The article satisfies the criteria outlined above; nevertheless, several points require further consideration before it can be published.

• Overall, the text is well written, presenting a clear and well-developed argument throughout. In the introduction, when referencing the literature, it would be helpful to briefly outline the contributions of the cited works, to ensure the references are not perceived as vague or lacking context.*

Response: We would like to thank the reviewer for their time reviewing our manuscript and these positive comments. The point regarding our referencing in the introduction is well-taken. We have now expanded the second paragraph of the introduction to clarify the main contributions of the references we had previously referred to only generally by now classing them into the different effects they examine and the conflicting findings found in some.  *• Using lagged effects—where mobility and policies respond immediately to changes in the COVID-19 growth rate, but influence cases only after a certain time period—contributes to mitigating the endogeneity problem. However, the system of equations is ultimately estimated in isolation, without incorporating a selection bias correction term or an instrumental variable. Instead, separate equations are estimated using lags to capture the effects. It would strengthen the econometric approach to establish a more cohesive relationship among these elements.*

Response: We agree in principle with the suggestion to use an instrumental variable approach to strengthen the causal identification strategy. Unfortunately, however, we are not aware of a variable that will satisfy the conditions for a valid instrument in this empirical setting. For our identification strategy to work, the change in policy restrictions needs to be ‘as random’, conditional on the covariates included, from which the variables capturing the state of the pandemic are most important. Indeed, as the reviewer notes, the lag structure of the effects is our approach to address endogeneity concerns, following the pioneering work of econometricians, such as Chernozhuhov et al. (2021). Our approach falls short of modelling an integrated system of equations, and we have removed references in the text that imply this. We have included a sentence in the conclusions that outlines these limitations of our approach.

*• The first part of the results explains restriction policies as a function of reported COVID-19 cases and deaths. However, it is not entirely clear how the results from the first part are incorporated into the second. If mobility depends on policy, and policy is determined by cases and deaths, there should be some temporal structure that allows these effects to be observed. It would be important to clarify how the use of lags fully addresses this issue. A first-stage estimation might be necessary to properly incorporate these effects into the model.*

Response: We agree that the first part of the analysis (policy restrictions as a function of cases and deaths) was confusing in its relationship to the rest of the analytical framework. Its purpose was only to confirm that policy follows in predictable ways from the changes in the course of the pandemic. We have therefore relegated this part to the Supplementary Information.

*• What explains the variation in the coefficients of the change in mobility with respect to excess mortality when the number of lag weeks is altered? A discussion of the results is crucial for understanding the optimal duration of the policy.*

Response: This is indeed an important question. Part of the answer has to do with the contemporarious correlations between the policy variable and the mobility variables, which are very high (between 0.48 and 0.82 in absolute size). When the policy variable is excluded from the equations, the coefficients for mobility are all in the expected direction (positive for presence in work, transport, retail and highly statistically significant): see the new version of Fig 4. When they are included together in the models, there is no sufficient independent variation in policy and mobility for both effects to be estimated precisely at the same time. Coupled with the autocorrelations of the mobility variables, this could account for the patterns we could see in the previous version of Fig 4, which showed the partial effects of mobility, *conditional on policy being in the model.* Unfortunately, in the absence of suitable instruments, we are not aware of a method that would allow us to estimate both the policy and mobility effects precisely. This is also why we only provide estimates of the ‘one-step-ahead’ effects of policy and of mobility, rather than statements about the optimal duration of policy or its cumulative effects on excess mortality over the full course of the pandemic.
